# High burden and multi-parasite profile of gastrointestinal infections in cattle from Limpopo District, Southern Mozambique: Epidemiology, risk factors, and One Health implications

**DOI:** 10.14202/vetworld.2025.3994-4008

**Published:** 2025-12-23

**Authors:** Edvânia Celso Manave, Guido André Nchowela, Avelino Raimundo Miguel, Carcésia César Matuassa, Aly Salimo Muadica, Benedito Machanja, Lúcel Fernandes, Omar Manito Mavilingue, Iúnice Simbine, Priscília Tsamba, Ilídio Filipe Manuel, Izaidino Jaime Muchanga, Taís Deta, Elina Manuel Ualema, Helder Cortes, Célio Alfredo

**Affiliations:** 1 Department of Veterinary Medicine, Faculty of Veterinary Medicine and Animal Science, Save University, National Road No. 1, Parcel 76, 1200 Chongoene, Gaza Province, Mozambique; 2 Faculty of Health Sciences, Zambezi University, Provincial Hospital, Josina Machel Neighborhood, Tete City, Mozambique; 3 Centro de Investigação, Instituto Português de Oncologia do Porto Francisco Gentil, E. P. E. Rua Dr António Bernardino de Almeida, 4200-072, Porto, Portugal; 4 Department of Forestry Engineering, Faculty of Agronomy and Forestry Engineering, Zambezi University, Nacogolone Campus, National Road No. 1, 2403 Mocuba City, Zambezia Province, Mozambique; 5 Herpetology Laboratory, Federal University of Santa Maria, Santa Maria, Rio Grande do Sul 97105-900, Brazil; 6 Faculty of Agricultural Sciences, Licungo University, Quelimane City, Mozambique; 7 Saint Thomas University of Mozambique, Praia Street, Xai-Xai City, Mozambique; 8 Department of Community Medicine, Information and Health Decision Sciences, Faculty of Medicine, University of Porto, Porto, Portugal; 9 Laboratório de Parasitologia Victor Caeiro, MED (Mediterranean Institute of Agriculture, Environment and Development), University of Évora, Évora, Portugal

**Keywords:** cattle, gastrointestinal parasites, Mozambique, One Health, prevalence, risk factors, tropical livestock epidemiology

## Abstract

**Background and Aim:**

Gastrointestinal (GI) parasites significantly affect cattle productivity and animal health, especially in tropical regions where environmental and management conditions favor parasite survival. In Mozambique, most previous studies have focused on goats or individual parasite species, leaving crucial gaps in understanding multi-parasite burdens in cattle. This study aimed to determine the prevalence, diversity, and risk factors associated with GI parasites in cattle from the Limpopo district of southern Mozambique, applying a One Health lens due to the zoonotic potential of some parasites that circulate in cattle.

**Materials and Methods:**

A cross-sectional study was conducted from January to May 2025 using 200 stool samples collected directly from cattle rectums. Samples were examined using Ritchie centrifugal sedimentation for helminths and protozoa and Ziehl–Neelsen staining for *Cryptosporidium* spp. Epidemiological data on grazing areas, deworming practices, and animal demographics were collected to identify risk and protective factors through Fisher’s test and odds ratios (OR).

**Results:**

Overall, 88.5% of cattle harbored at least one GI parasite. Eight parasite groups were detected: Eimeria spp. (49%), Strongyle-type eggs (46.5%), ciliates (29.5%), *Paramphistomum* spp. (18%), Fasciola spp. (11%), Cryptosporidium spp. (3.5%), Giardia spp. (2.5%), and *Entamoeba* spp. (1.5%). Grazing in non-flooded areas significantly reduced Fasciola spp. infection. (OR = 0.126) and *Paramphistomum* spp. (OR = 0.236), whereas deworming reduced Strongyle-type infections (OR = 0.366). Conversely, dewormed animals had higher odds of Eimeria spp. and ciliate infections, likely because ivermectin was ineffective against protozoa. Co-infections were common, particularly among adult animals.

**Conclusion:**

This first multi-parasite epidemiological assessment in Mozambican cattle reveals a high burden of GI parasites, influenced by grazing conditions and suboptimal deworming practices. Avoiding flooded grazing areas, adopting coccidiostats, and implementing anthelmintic rotation are crucial for effective parasite control. Given the zoonotic relevance of *Cryptosporidium*, *Giardia*, and *Fasciola*, molecular studies are urgently needed to characterize circulating species and clarify the role of cattle as reservoirs. These findings provide essential evidence to strengthen veterinary surveillance and inform One Health interventions in southern Mozambique.

## INTRODUCTION

Gastrointestinal (GI) parasites, including nematodes, trematodes, cestodes, and protozoa, infect cattle and have direct implications for animal health and welfare. These infections jeopardize herd productivity by reducing weight gain and carcass size, lowering feed intake and growth rates, and negatively affecting fertility and milk production [1, 2]. Many infections occur subclinically, complicating early diagnosis and hindering the timely implementation of control measures [1]. The effects of these parasites are particularly severe in developing regions such as sub-Saharan Africa, where challenges related to sanitation, grazing systems, and food management are widespread [3]. Among the parasites commonly identified in cattle, *Strongyloides*, Strongyle-type parasites, *Fasciola*, *Paramphistomum*, *Moniezia*, *Toxocara*, *Trichuris*, and *Coccidia* are frequently detected in stool samples [4, 5].

Helminthic infections can lead to metabolic disorders, emaciation, and increased vulnerability to secondary pathogens, whereas coccidial infections may result in diarrhea, dehydration, dysentery, debilitation, and even death, especially in young or heavily parasitized cattle [6]. Fasciolosis represents one of the most important parasitic diseases affecting cattle in tropical and subtropical regions, where environmental conditions favor transmission of *Fasciola* spp. [1]. Beyond its economic impact, fascioliasis is also a growing zoonotic concern, reported increasingly in regions such as the Middle East and North Africa [7]. Infection with *Fasciola* spp. contributes to reduced growth rates, decreased milk and meat production, liver condemnation at slaughter, and heightened susceptibility to secondary infections [8]. Studies worldwide have shown substantial variation in the prevalence of *Fasciola* spp. in cattle, influenced by environmental factors, management practices, and the abundance of intermediate snail hosts. For instance, in Burundi, a prevalence of 47.7% was reported based on 426 stool samples [9], while in São Paulo, Brazil, *Fasciola* infection was identified in 45.6% of 1,450 analyzed samples [1].

Although GI parasitism is widely recognized as a major constraint to cattle health and productivity in tropical regions, the existing body of research in Mozambique remains notably fragmented and species-restricted. Most available studies have focused predominantly on goats, as shown by Atanasio-Nhacumbe and Sitoe [10], who documented nematode prevalence and seasonal variations in small ruminants from Tete and Cabo Delgado provinces. In contrast, equivalent investigations in cattle are virtually absent, with the exception of a single study conducted in the Magude district, where Miambo *et al*. [11] examined only two protozoan agents, *Cryptosporidium* and *Giardia*, using limited diagnostic techniques that did not account for the full spectrum of helminths, trematodes, or cestodes relevant to bovine health. Additionally, routine surveillance data from the Ministry of Agriculture and Rural Development [12] report Fasciola occurrences only at the provincial level, without diagnostic confirmation, epidemiological characterization, or risk factor assessment. Collectively, these sources underscore a critical knowledge gap: Mozambique lacks any comprehensive, multi-parasite, sample-based epidemiological study in cattle that evaluates parasite diversity, co-infections, and environmental or management-related determinants. This gap limits the development of targeted control programs and constrains national understanding of zoonotic risks associated with cattle parasitism. Addressing this deficiency is essential to strengthening veterinary public health strategies and advancing a One Health perspective in a region where parasitic diseases have historically been under-investigated.

Given the limited and fragmented information available on GI parasites in cattle in Mozambique, particularly the absence of comprehensive, multi-parasite investigations that analyze epidemiological patterns, co-infections, and associated risk factors, this study aimed to conduct the first broad parasitological assessment of cattle in the Limpopo district of southern Mozambique. Specifically, the study sought to determine the prevalence and diversity of key helminths, trematodes, cestodes, and protozoa circulating in local herds, while identifying animal-, environmental-, and management-related factors that influence infection dynamics. By integrating parasitological diagnosis with contextual production data and interpreting the findings through a One Health lens, this study aims to provide evidence needed to strengthen parasite control programs, improve livestock productivity, and clarify potential zoonotic risks relevant to both animal and human health in a historically understudied region.

## MATERIALS AND METHODS

### Ethical approval

This research was approved by the Scientific Directorate of the Faculty of Veterinary Medicine and Zootechnics, Save University, and by the District Services for Economic Activities of the Limpopo district (reference numbers 03/MFVZ/UniSave/2025 and 06/SDAE Limpopo/2025, respectively). All livestock farmers received a consent form, which was read in Portuguese and translated into the local language. Stool samples were collected only after a family representative signed the form and could write their own name. The fieldwork was conducted in accordance with the standards set out in the Mozambique Decree on Animal Health Regulations, approved in 2009 [13].

In line with the Animal Research: Reporting of *In Vivo* Experiments (ARRIVE) 2.0 guidelines, all methods, procedures, and reporting were conducted to ensure transparency, reproducibility, and compliance with internationally accepted animal welfare principles. Sample collection was limited to rectal retrieval of feces, a non-invasive, minimally disruptive procedure that does not cause harm, pain, or lasting distress to the animals. No experimental treatments, interventions, sedation, or procedures outside routine handling were performed, and animals were restrained only briefly using standard husbandry practices to ensure operator and animal safety.

Animal welfare was monitored continuously throughout sampling, and no adverse events were observed. Participation by farmers was voluntary, and all personal and farm data were recorded, stored, and analyzed in a confidential manner, complying with ethical expectations for field-based veterinary epidemiological studies.

### Study period and location

**Figure 1:**
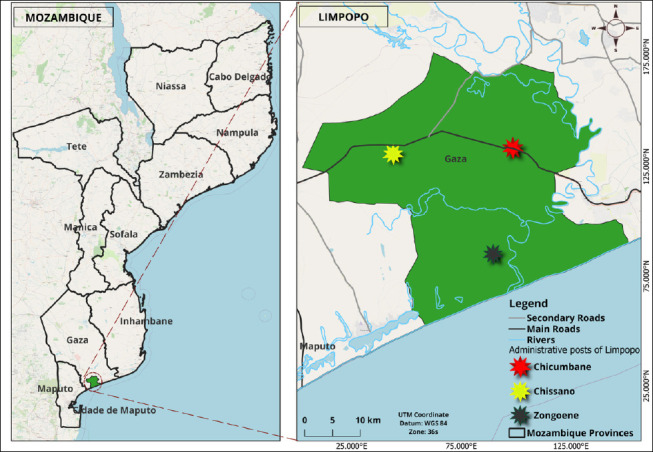
Geographical location of Limpopo district and administrative posts [Source: This map was generated using the software *QGIS*, version 3.40].

The study was conducted from January to May 2025 in the Limpopo district, located in Gaza province in southern Mozambique (Figure 1). The district is located approximately between coordinates 24°39′S and 33°24′E. The district is divided into three administrative posts: 1) Chissano, which includes the localities of Chikotane, Chimonzo, Chissano, and Licilo; 2) Chicumbane, which includes the localities of Chicumbane, Chiridzene, Languene, Muamuasse, Muawasse, Muzingane, and Nuvunguene; and 3) Zonguene, which includes the localities of Chilaulane, Nhambanga, Novela, and Zonguene [14].

The predominant climate in the region is tropical dry, characterized by average annual temperatures of 22°C–26°C and a relative humidity of 60%–65%. The annual rainfall is low and, combined with the high temperatures, results in a marked water deficiency, contributing to droughts and dry spells even during the rainy season. The region’s soil is predominantly sandy, with alluvial deposits along the Limpopo River, which directly influences the district’s fertility and agricultural suitability [15].

According to data from the Ministry of Agriculture and Rural Development, Limpopo has an estimated cattle population of around 29.571 [12]. Local animal production, as in other districts in the province, predominantly follows a traditional, extensive system, with small- and medium-sized production units emphasizing beef cattle farming. This activity plays a crucial role in communities’ subsistence, as ruminant farming is a major source of wealth and livelihood [16].

In Limpopo, as in the other districts of Gaza province, the difference between the dry and rainy seasons is less marked; the rainy season runs from October to May and is characterized by hot humid conditions [17, 18].

### Study design and sampling methods

A cross-sectional epidemiological study with random sampling was conducted. To achieve this goal, 57 small-scale, extensive cattle producers were visited, with a predominance of the Landim breed, which is exclusively raised for meat production. Grazing is conducted in shared pasture areas and close to water sources.

A total of 200 stool samples were collected, with 3-4 samples from each family sector, categorized as follows: animals were classified as juvenile (up to 3 years) or adult (> 3 years), as applied in the study conducted in Nigeria [19]. However, due to the small number of calves and yearlings in the study area, these two groups were combined into a single category to enhance statistical power.

Initially, a sample size of 343 animals was estimated and distributed between Limpopo and Mabalane districts in the province of Gaza. This number was calculated using the formula proposed by Thrusfield [20], with an expected prevalence of 50%, a confidence level of 95%, and a margin of error of 0.05. However, due to financial constraints, sampling campaigns in the Mabalane district were not possible, limiting the study to 200 samples collected in Limpopo.

Before sampling, local veterinary service authorities were consulted to obtain a list of administrative posts and the respective cattle population at each point. The three administrative posts in the district were included to ensure representativeness. At each post, specific locations were selected based on the availability of restraint chutes to facilitate direct rectal collection of stool, ensuring the safety of both the operator and the animals. Thus, in the administrative post of Chicumbane, which has the largest herd (16,366 animals), the localities of Mowawaze, Muzingane, and Chibandzene were selected. In Chissano, with 9,625 animals, only the Chicotane location was included, as it was the only one with a chute available. Finally, the Novela location in Zongoene, with 3,580 animals, was selected.

The breeders were made aware of the situation and invited to take their animals to the collection points. Stool samples were collected from small farms with 1-99 animals. Samples could not be obtained from medium-sized farms with 100-500 animals or large farms with more than 500 animals due to the absence or low frequency of this breeder in the region. Individuals were selected by stratified random sampling: first, the herds were divided into strata according to sex and age group. Then, the animals were chosen randomly within each stratum by assigning numbers to the collars and subsequently drawing lots/generating random numbers. This methodology ensured proportionality between males and females, as well as between young and adult animals, reflecting the actual structure of the herds.

At the three administrative posts, females outnumbered males, and there were more adult than young animals. In addition, a survey was conducted to collect information on management conditions, including grazing area characteristics, deworming practices, breeding systems, and herd size.

### Stool collection and analysis

The study included 200 stool samples from meat-producing animals. The stool samples were collected from random cattle, directly from the rectum, using latex gloves, in quantities ranging from 10–30 g. During sample collection, hygiene measures were implemented by systematically changing gloves for each animal to prevent cross-contamination.

After collection, the samples were duly identified, packed in isothermal boxes with ice accumulators, no added preservatives, and transported immediately to the general laboratory of the Chongoene Health Center, parasitology sector, for concentration and primary analysis, and to the biology laboratory at Save University, to perform the staining of microscope slides. The time elapsed between sample collection and processing was approximately 8 h, with processing performed immediately on the same day. Laboratory processing was performed using two techniques: (1) the Ritchie technique (sedimentation by centrifugation) to detect cysts and light and heavy eggs, and (2) Ziehl–Neelsen staining to detect oocysts of *Cryptosporidium* spp.

### Centrifugal sedimentation

To perform the Ritchie technique [21], approximately 3 g of the stool sample was homogenized in 15 mL of distilled water and filtered through hydrophilic gauze. The collected material was transferred to a graduated conical tube, distilled water was added to make up a volume of 14 mL, and the mixture was centrifuged in a Thermo Scientific Megafugi 8R centrifuge (USA) at 1,000 × *g* for 5 min, repeating the process until the supernatant was clear.

After centrifugation, 10 mL of distilled water and 4 mL of ether were added to the tube, and the mixture was shaken vigorously for 10 s to ensure complete homogenization. The tube was then centrifuged again under the same conditions, separating the contents into four layers: ether, residue, water, and sediment at the bottom. The supernatant was carefully discarded. The obtained sediment was mixed, and an aliquot was transferred to a microscope slide covered with a coverslip.

The cysts, oocysts, and eggs of the parasites were identified based on their morphological characteristics using an optical microscope (Motic, model BA310, China) with 10× and 40× objectives, allowing detailed observation of the parasite structures [22]. The observed parasitic structures were compared with images of parasites from veterinary parasitology charts obtained from the Department of Veterinary Medicine at the University of Évora, Portugal.

### Ziehl–Neelsen staining

For the specific detection of *Cryptosporidium* spp., stool smears were obtained from the sediment using the Ritchie technique, spread on microscopy slides, and dried at room temperature (37°C) [23]. The slides were then fixed with methanol for 30 s and stained with carbol fuchsin for 1 min. After staining, the slides were washed with distilled water, soaked in an acid-alcohol solution for 2 min, then washed again with distilled water. Malachite green was applied for 2 min, and the slides were washed and dried at room temperature.

To visualize *Cryptosporidium* spp. oocysts, the samples were observed under light microscopy (Motic, model BA310) using an immersion objective (100x). The slides were read by two independent individuals and confirmed by a third microscopist. Due to difficulties in obtaining reference slides in the region, the identification of *Cryptosporidium* spp. oocysts was based on veterinary parasitology slides provided by the Department of Veterinary Medicine, University of Évora, Portugal, where the parasite appears pink to red in color, spherical to ovoid in shape, with bodies and a bluish background [24].

### Statistical analysis

All animal data and laboratory results were recorded on the forms designed for this purpose and then digitized in Microsoft Excel on Windows 10. In a third stage, all the information was exported to the Statistical Package for the Social Sciences (SPSS Inc., Chicago IL, USA) program, version 23.0, to estimate the prevalence of GI parasites, as well as cross-referencing the data with socio-demographic variables of the production units, such as: age, gender, frequency of deworming, grazing areas, and animal category. Fisher’s test and the odds ratio (OR) were used to determine the main factors associated with the cattle infection by the enteroparasites considered in this study, adopting a significance level of 0.05.

Descriptive statistics were used to assess the rates of cattle coinfection with various parasites. Univariate analysis was used to explore the direct associations between each independent variable and the outcome.

## RESULTS

### Overall prevalence of GI parasites

**Figure 2:**
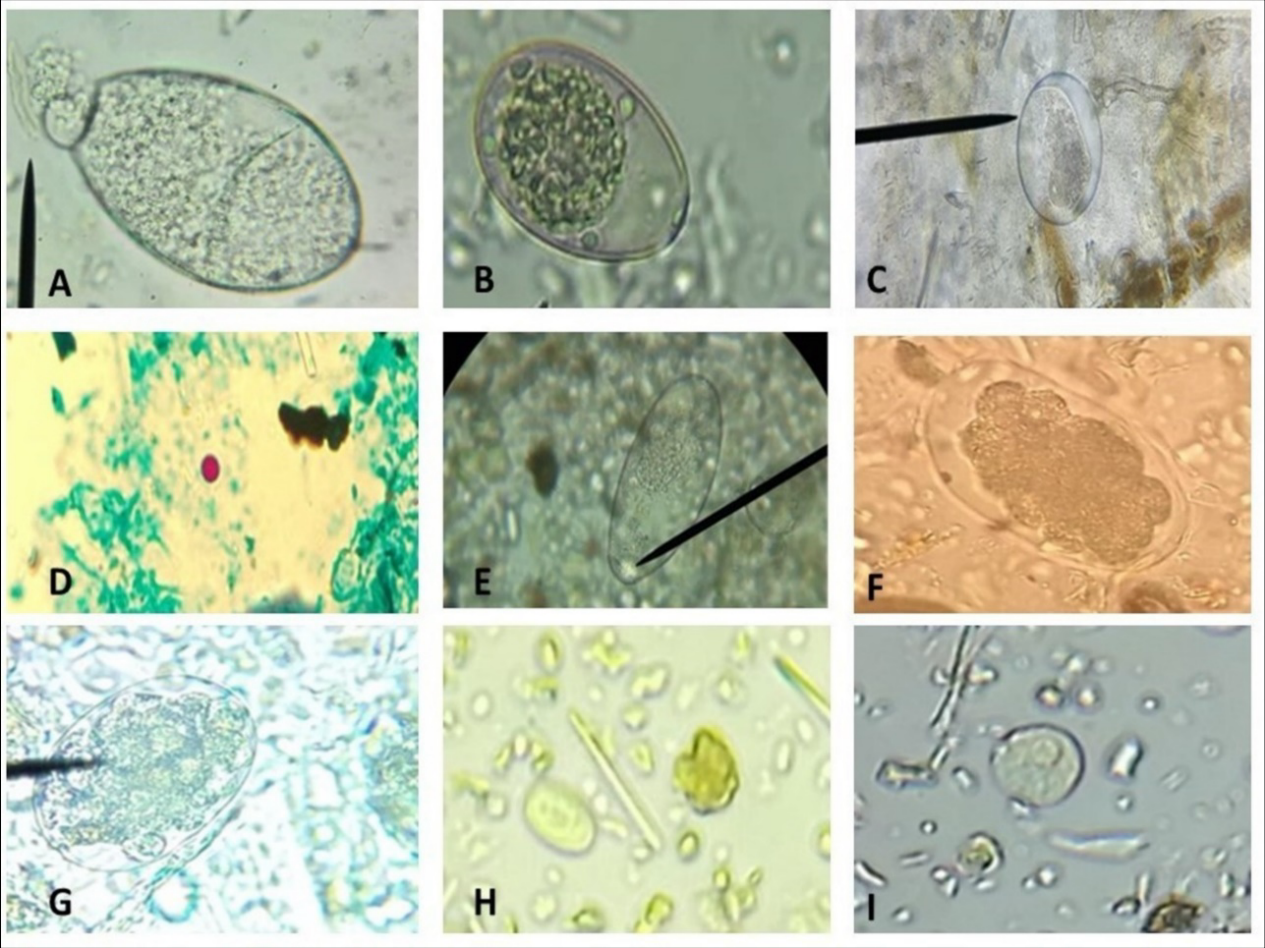
Cysts, oocysts, and parasite eggs detected in stool samples from cattle in Chongoene district, Gaza province, southern Mozambique. (A) *Fasciola* spp. egg (Ba r= 55 μm), (B) *Eimeria* spp. oocyst (Bar = 30 μm) (C) Ciliate cyst (Bar = 30 μm), (D) *Cryptosporidium* spp. oocyst (1000X), (E) *Paramphistomum* spp. egg (Bar = 50 μm ), (F and G) Strongyle-type eggs (Bar = 65 μm), (H) *Giardia* cyst (Bar = 10 μm), and (I) *Entamoeba* spp. cyst (Bar = 10 μm).

In this study, 200 samples from cattle raised in three administrative posts of the Limpopo district in southern Mozambique were analyzed. It was found that 88.5% (177/200; 95% confidence interval [CI] 84.1–92.9) of the samples contained at least one or more GI parasites. A total of eight different parasites were detected, namely: *Paramphistomum* spp., *Fasciola* spp., *Eimeria* spp., Strongyle-type, Ciliate cysts, *Entamoeba* spp., *Giardia* and *Cryptosporidium* spp., of which *Eimeria* was the most prevalent parasite at 49% (98/200) (95% CI 42.1–55.9), followed by Strongyle-type 46.5% (93/200) (95% CI 39.6–53.4), Ciliate cysts at 29.5% (59/200) (95% CI 23.2–35.8), *Paramphistomum* spp. at 18% (36/200) (95% CI 12.7–23.3), *Fasciola* spp. at 11% (22/200) (95% CI 6.7–15.3), *Cryptosporidium* spp. at 3.5% (7/200) (95% CI 1.0–6.0), *Giardia* 2.5% (5/200) (95% CI 0.3–4.7) and finally *Entamoeba* spp. with 1.5% (3/200) (95% CI 0.0–3.2), as illustrated in Table 1 and Figure 2.

**Table 1:** Prevalence of gastrointestinal parasites in cattle in the Limpopo district, Gaza province, southern Mozambique.

Parasite	Positive (n)	Prevalence (%)	95 Confidence interval
Strongyle-type	93	46.5	39.6–53.4
*Eimeria* spp.	98	49	42.1–55.9
*Paramphistomum* spp.	36	18	12.7–23.3
*Fasciola* spp.	22	11	6.7–15.3
Ciliates	59	29.5	23.2–35.8
*Giardia*	5	2.5	0.3–4.7
*Cryptosporidium* spp.	7	3.5	1.0–6,0
*Entamoeba* spp.	3	1.5	0.0–3.2

### Distribution of parasites across administrative posts

Comparing the cattle infections among the administrative posts (Table 2), the highest rate of enteroparasite infection was observed in Chissano, with 96% (24/25), 95% CI 88.4–100 of cases, followed by Chicumbane, with 91.3% (115/126), 95% CI 86.4–96.2, and Zongoene, with 77.6% (38/49), 95% CI 65.8–89.4. Differences in the distribution of infections between the posts were statistically significant (p = 0.015).

**Table 2:** Prevalence of gastrointestinal parasites in cattle among the administrative posts from Limpopo district, Gaza province, southern Mozambique.

Administrative post	Total samples	Negative	Positive	Prevalence	95% Confidence interval	p-value
Chicumbane	126	11	115	91.3	86.4–96.2	0.015
Zongoene	49	11	38	77.6	65.8–89.4	
Chissano	25	1	24	96	88.4–100	

In Chicumbane, 40.8% (51/126) of the samples were positive for Strongyle-type eggs, 7.9% (10/126) for *Fasciola* spp., 14.3% (18/126) for *Paramphistomum* spp., 56.3% (71/126) for *Eimeria* spp., 45.2% (57/126) for Ciliate cysts, 3.9% (5/126) for *Giardia*, 2.4% (3/126) for *Entamoeba* spp. cysts, and 5.6% (7/126) for *Cryptosporidium* spp.

For the administrative post of Zongoene, the following infection rates were observed for each parasite: *Fasciola* spp., with 12.2% (6/49), *Paramphistomum* spp., 10.2% (5/49), Strongyle-type 55.1% (27/49), *Eimeria* spp., 38.8% (19/49) and Ciliate cysts 4.1% (2/59), with no parasites such as *Giardia*, *Entamoeba* spp., and *Cryptosporidium* spp. The parasites detected in Chissano were as follows: *Fasciola* spp., 24% (6/25); *Paramphistomum* spp., 52% (13/25); Strongyle-type, 60% (15/25); and *Eimeria* spp., 32% (8/25) (Supplement 1).

We observed that the prevalence of parasites was higher in females, with 54% (108/200), than in males, who had 34.5% (69/200); however, this difference was not statistically significant (p = 0.23). Similarly, adult animals had a higher infection rate (50.5%; 101/200) than young animals (38%; 76/200), without a statistically significant difference (p = 0.45).

### Animal practices and environmental conditions

When comparing the prevalence of parasites and deworming practices, 67.5% (135/200) of animals did not receive regular antiparasitic treatment, whereas 21% (42/200) received regular deworming. However, this difference was not statistically significant (p = 0.49).

We observed that infection by *Fasciola* spp. (21.9%; 18/82) and *Paramphistomum* spp. (30.5%; 25/82) was more frequent in flooded areas than in non-flooded areas, with prevalence rates of 3.4% (4/118) and 9.3% (11/118), respectively. In contrast, Strongyle-type and *Eimeria* spp. showed a higher frequency in non-flooded areas, with 45.8% (54/118) and 61.9% (73/118), respectively, compared to flooded areas, where the prevalences were 47.5% (39/82) for Strongyle-type and 30.9% (25/82) for *Eimeria* spp. The observed differences were statistically significant (p = 0.01).

### Patterns of coinfection

**Figure 3:**
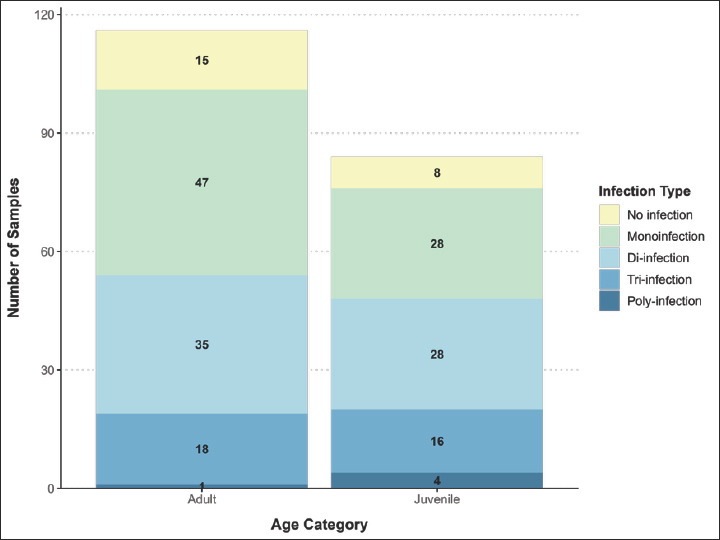
Coinfection pattern of the samples according to the age of the animals.

The coinfection rate varied by animal category, with higher rates in adults than in young animals. In adult animals, most samples (47, 40.5%) were infected with a single parasite, and only one sample (0.9%) was infected with multiple parasites. In young animals, the rates of coinfection with two parasites were the same as those of monoinfection: 28 (33.3%), 16 (19.0%) had triple infection, and 4 (4.8%) had polyinfection (Figure 3).

### Risk factors associated with GI infection

As shown in Table 3, the variables “deworming frequency,” “pasture location,” “farm location,” and “animal category” served as risk or protective factors for GI parasite infections detected in this study. However, no statistically significant association was found between the variable “sex of the animals” and the presence of any parasite analyzed.

**Table 3:** Risk and protective factors for gastrointestinal parasite infection in cattle in the Limpopo district, southern Mozambique.

Parasite	Variable	Risk factor	OR	95 CI	Global p-value
Strongyle-type	Gender	Female Ref			0.533
		Male	1.20	0.65–2.22	
	Deworming	Yes	0.37	1.28–5.87	0.004
		No Ref			
	Grasslands	Flooded Ref			0.802
		Not flooded	0.93	0.51–1.70	
	Administrative posts	Zongoene Ref			0.077
		Chicumbane	0.68	0.32–1.48	
		Chissano	1.51	0.41–5.52	
	Category	Juvenile Ref			0.006
		Adult	0.37	0.20–0.68	
*Paramphistomum* spp.	Gender	Female Ref			0.849
		Male	0.93	0.40–2.08	
	Deworming	Yes	1.30	0.50–3.70	0.575
		No Ref			
	Grasslands	Flooded Ref			0.001
		Not flooded	0.24	0.10–0.54	
	Administrative posts	Zongoene Ref			0.001
		Chicumbane	0.17	0.08–0.36	
		Chissano	1.08	0.32–3.72	
	Category	Juvenile Ref			0.245
		Adult	1.56	0.70–3.67	
*Fasciola* spp.	Gender	Female Ref			0.907
		Male	0.95	0.33–2.57	
	Deworming	Yes	3.23	0.75–33.3	0.100
		No Ref			
	Grasslands	Flooded Ref			0.003
		Not flooded	0.13	0.03–0.40	
	Administrative posts	Zongoene Ref			0.060
		Chicumbane	0.09	0.03–0.21	
		Chissano	0.32	0.07–0.41	
	Category	Juvenile Ref			0.729
		Adult	0.85	0.32–2.33	
*Eimeria* spp.	Gender	Female Ref			0.092
		Male	0.61	0.33–1.13	
	Deworming	Yes	2.09	1.02–4.42	0.029
		No Ref			
	Grasslands	Flooded Ref			0.001
		Not flooded	3.67	1.95–7.06	
	Administrative posts	Zongoene Ref			
		Chicumbane	1.29	0.48–3.51	0.021
		Chissano	0.47	0.14–1.50	
	Category	Juvenile Ref			0.598
		Adult	0.86	0.47-1.57	
Ciliate cysts	Gender	Female Ref			0.357
		Male	1.34	0.68–2.60	
	Deworming	Yes	4.17	1.98–8.91	0.002
		No Ref			
	Grasslands	Flooded Ref			0.002
		Not flooded	16.81	5.75–67.40	
	Administrative posts	Zongoene Ref			0.001
		Chicumbane	0.83	0.37–1.87	
		Chissano	-	-	
	Category	Juvenile Ref			0.945
		Adult	0.98	0.51–1.91	

In the specific case of *Fasciola* spp. and *Paramphistomum* spp., among the five variables assessed, the only statistically significant association was observed with the variable “location of grazing areas” (p = 0.00003; OR = 0.126; p = 0.0001; OR = 0.236). These results indicate that grazing in non-flooded areas acts as a protective factor against both species’ infection. Infection by *Paramphistomum* spp. showed significant variation depending on the administrative post (p = 0.00001), with a higher risk of infection being observed in Chissano (OR = 1.083).

In the case of Strongyle-type, significant relationships were identified in the variables related to frequency of deworming (p = 0.004, OR = 0.366) and animal category (p = 0.0006, OR = 0.369), indicating that deworming cattle acts as a protective factor, making dewormed animals 63% less likely to contain this parasite. The same probability was observed in adult animals, which are less likely to be infected than young animals.

Furthermore, ciliate infection also varied by administrative post and was significantly higher in the Chicumbane group (p = 0.000000001). In contrast, no statistically significant associations were found between all the variables considered in this study and the parasites *Giardia* spp., *Entamoeba* spp., and *Cryptosporidium* spp. due to the small number of positive cases, which limited the assessment of statistical associations.

## DISCUSSION

### Overall burden of GI parasites

In this study, the overall prevalence of GI parasites in cattle in the Limpopo district was 88.5%, which is one of the highest infection rates observed in southern Africa. This study used low-cost, reliable methods that can be adapted for use in veterinary laboratories with limited resources and that provide important data on the epidemiology of enteroparasites. This rate reflects a multifactorial interaction between climatic conditions, inadequate management practices (irregular deworming, grazing in flooded areas), and limited access to veterinary services, which are common in Limpopo. These findings are consistent with those of studies conducted in similar tropical contexts. In Ghana, for example, a prevalence of 90.85 % was reported [25], and in Ethiopia, specific prevalences of 50% and 36% for GI strongyles and *Coccidia*, respectively [26]. These data reinforce the recurring pattern of high infection rates in tropical regions with extensive or semi-intensive cattle production systems. Nevertheless, considerably lower prevalence rates have been observed in some regions of the world. Studies conducted in Uganda and Pakistan reported prevalences of 32.2% and 33.68%, respectively [27, 28]. These variations may be associated with differences in production systems, ecological conditions, sanitary practices, and parasite control strategies adopted in each location.

### Prevalence of *Eimeria* and Strongyle-type parasites

Among the identified parasites, *Eimeria* spp. (49%) Strongyle-type (46.5%) was the most prevalent. These results corroborate the findings of Frias *et al*. [1], who reported prevalences of 39.8% for *Eimeria* spp. and 35.3% for Strongyle-type eggs in cattle in the state of São Paulo, Brazil. Similarly, in a study conducted in Ghana [25], these two groups were also identified as the most prevalent among six genera of parasites, with infection rates of 80.5% for *Eimeria* spp. and 65.9% for other GI parasites. The high prevalence of these parasites may be associated with the high environmental resistance of parasite oocysts and eggs, the lack of effective control strategies, such as non-rotation of pastures, inadequate use of antiparasitic drugs, prolonged exposure to humid environments that favor the survival of eggs and larvae, and the predominance of extensive rearing systems [1]. The recurrence of these parasites in different geographical and production contexts suggests that *Eimeria* spp. and Strongyle-type parasites are of significant epidemiological importance for cattle farming in tropical and subtropical regions.

### Occurrence and identification challenges of ciliate cysts

In this study, ciliate cysts were also detected in 29.5% of the analyzed samples. However, it was not possible to accurately identify these organisms at the genus or species-level. This limitation was due to the morphological similarity between the cystic forms of *Balantidium coli* and *Buxtonella sulcata*, both of which are known to infect cattle and have similar microscopic characteristics, which can compromise the accuracy of parasitological diagnosis [29]. This difficulty underscores the need for complementary techniques, such as molecular analysis, to achieve more accurate and reliable taxonomic identification of these protozoa.

### Prevalence and environmental associations of Fasciola and *Paramphistomum*

The overall prevalence of Fasciola spp. observed in this study (11%) is similar to that previously reported by the animal health authorities in Mozambique [12], which reported a prevalence of 16% for *Fasciola* spp. in Gaza province. Although this study shows a slight reduction in the percentage, more recent data [30] indicate an increase in infections, suggesting that transmission remains active in the region. This scenario is especially evident in wetlands, where prevalence reached 21.9%, reinforcing the association between *Fasciola* spp. and aquatic environments [31].

Similar to the findings for *Fasciola* spp., *Paramphistomum* spp. showed a significantly higher prevalence in flooded areas (30.5%) than in dry regions (9.3%). The statistically significant association between grazing animals in flooded areas and infection by *Fasciola* and *Paramphistomum* represents a discovery in the Limpopo District and Mozambique in general, and reinforces the need for animal health authorities to develop specific protocols to raise awareness and educate livestock farmers to avoid grazing animals in flooded areas.

The presence of aquatic mollusks, which act as intermediate hosts, is fundamental for the parasitic life cycle’s continuity. Ruminants become infected by ingestion of metacercariae in vegetation or contaminated water, especially in regions with waterlogged pastures or near waterbodies [31]. These findings reinforce the need for specific management measures in flooded areas to mitigate the risk of cattle trematode infections. Factors such as rainfall seasonality, pasture management, and access to flooded areas directly influence parasitic infection dynamics in cattle [32, 33].

### Low prevalence of protozoan parasites

In contrast, the protozoa *Giardia* spp. (2.5%), *Cryptosporidium* spp. (3.5%) and *Entamoeba* spp. (1.5%) showed considerably lower prevalences in this study. These findings are in line with those reported by Miambo *et al*. [11], who investigated the prevalence of these agents in calves in the district of Magude, southern Mozambique, using flotation (Willis), Ziehl–Neelsen staining, and immunofluorescence, and reported prevalence rates that varied by method. Similarly, reported low prevalences of these parasites in cattle using molecular methods in Indonesia, reinforcing the observed trend [34]. In addition, a study conducted in China [35] using polymerase chain reaction identified prevalence rates of 4.2% for *Cryptosporidium* spp. and 1% for *Giardia* spp., values close to those found in the present study. In a systematic review and meta-analysis, the global prevalence of Giardia was 24%, with wide variation across diagnostic techniques [36].

Despite their relatively low prevalence, these protozoa pose significant health risks, especially in regions with poor sanitation infrastructure [35, 37]. *Cryptosporidium* spp. are among the main etiological agents of diarrhea outbreaks in humans, while *Giardia* is associated with high morbidity in rural and urban areas [11, 35]. In Mozambique, epidemiological studies have reported human prevalences ranging from 9.7% to 41.7% for *Giardia* and 1.6% to 11.5% for *Cryptosporidium* [38–40]. This information highlights the need for an integrated One Health approach to control these agents through detection and molecular characterization to clarify the role of domestic animals as reservoirs.

### Spatial variation and administrative differences

The differences in prevalence among the administrative posts were statistically significant (p < 0.05), with Chissano showing the highest infection rate (96%), followed by Chicumbane (91.3%) and Zongoene (77.6%). The high prevalence observed in Chicumbane and Chissano may be related to various factors, including local environmental conditions, such as flooded areas and wet pastures, combined with inadequate management practices during the rainy season. The overload of pasture fields and irregular access to deworming programs may also have contributed to the higher parasite load. These factors underscore the importance of geographical context and management practices in modulating the prevalence of enteroparasites across locations.

### Influence of animal sex and physiological status

The analysis of prevalence by gender revealed a higher infection rate in females (54%) than in males (34.5%), although the difference was not statistically significant. This data should be interpreted with caution due to the larger number of females sampled. Despite the lack of significance in this study, the literature suggests that females, especially during pregnancy and lactation, may be more susceptible to parasitic infections due to reduced resistance [31]. Similar results were observed in Pakistan [28]. These findings indicate that the gender and physiological state of animals may modulate immune response, even in the absence of a statistical association.

### Influence of age on parasitic infection

Regarding age, a higher infection rate was observed in adults (50.5%) than in juveniles (38%), although the difference was not statistically significant. The farm demographics, with a predominance of adults, may have influenced this finding. While the literature commonly links greater susceptibility in juveniles to immature immunity [1, 10, 31, 41, 42], other studies report higher infection rates in adults [43, 44]. This variability highlights the complexity of parasitic epidemiology and indicates that age alone cannot explain infection patterns across systems.

### Impact of deworming practices and anthelmintic use

The prevalence of parasites was significantly higher in animals not regularly dewormed (67.7%), indicating that the absence of effective deworming practices favors reinfection and the persistence of parasite cycles. Studies conducted in Ghana [25] and Pakistan [28], corroborate these findings. Inadequate practices, incorrect drug administration, and lack of continuous diagnosis contribute to anthelmintic resistance [1, 45]. The analysis of risk factors supported this relationship: regular deworming reduced the likelihood of Strongyle-type infection by 63%.

Curiously, for *Eimeria* spp. and ciliates, deworming was associated with higher prevalence, suggesting it is a risk factor for these parasites. This can be explained by the predominant use of ivermectin, which is ineffective against protozoa [31, 46]. Evidence from studies in translocated Woylies demonstrates that ivermectin reduces helminths but not *Coccidia* [47]. This highlights how anthelmintic-based control programs may overlook protozoa in Limpopo. Therefore, authorities must consider updating protocols, especially regarding anticoccidiostats and drug rotation [46, 48].

### Clinical and public health importance of identified parasites

From a clinical perspective, the parasites identified in this study have variable impacts on animal and public health. Strongyle-type parasites cause anemia, weight loss, and reduced productivity [49]. *Eimeria* spp. cause coccidiosis, especially in young animals. *Fasciola* spp. and *Paramphistomum* spp. compromise liver and rumen function, respectively [46].

Protozoa such as *Giardia*, *Cryptosporidium*, and *Entamoeba* spp., though at low prevalence, pose zoonotic risks, especially in immunocompromised individuals or in areas with poor sanitation [35]. The prevalence of 11% for *Fasciola* spp. also indicates economic losses through liver condemnation and reduced performance, a significant zoonotic threat [50], and is considered an emerging disease for humans in many tropical regions [50, 51].

Thus, this is the first study conducted in the Limpopo district to detect multiple parasites in a single epidemiological survey. This study provides a more holistic overview of parasitic infections in this region and facilitates the development of more effective zoonosis control strategies aligned with regional risk factors.

### Study limitations

Although the study provided relevant epidemiological data on the prevalence of GI parasites in cattle, it was limited by its reliance solely on parasitological methods, which are less sensitive and specific than molecular methods. The application of molecular methods could help identify *Cryptosporidium* spp. species and *Giardia duodenalis* variants, as well as differentiation between *B. coli* and *B. sulcata*. Some reports provided by cattle breeders on deworming practices may have been influenced by forgetfulness.

## CONCLUSION

This study provides the first comprehensive epidemiological assessment of GI parasites in cattle in the Limpopo district of southern Mozambique, revealing an exceptionally high overall prevalence of 88.5%. Eight parasitic groups were detected, with *Eimeria* spp. (49%) and Strongyle-type nematodes (46.5%) being the most dominant, followed by substantial occurrences of ciliate cysts (29.5%), *Paramphistomum* spp. (18%), and *Fasciola* spp. (11%). The spatial differences observed between administrative posts, along with the strong associations between parasite prevalence and environmental characteristics, particularly grazing in flooded areas, underscore the influence of agro-ecological conditions on transmission dynamics. Although protozoa such as *Giardia*, *Cryptosporidium*, and *Entamoeba* spp. were detected at lower frequencies, their presence highlights important zoonotic considerations in a region already burdened by limited sanitation and high vulnerability to waterborne diseases.

From a practical standpoint, the findings demonstrate the urgent need to improve parasite management strategies in Limpopo. Irregular deworming, reliance on a single anthelmintic (ivermectin), grazing in waterlogged areas, and limited access to veterinary services all contribute to sustained transmission. The detection of *Fasciola* and *Paramphistomum* infections in flooded grazing systems emphasizes the need for targeted farmer education, pasture management interventions, and the incorporation of snail-control strategies or seasonal grazing adjustments. For protozoan parasites, the results highlight the necessity to integrate diagnostic methods capable of distinguishing between morphologically similar species and identifying zoonotic strains to inform One Health interventions.

A major strength of this study lies in its multi-parasite diagnostic approach, broad sampling coverage across three administrative posts, and the use of cost-effective laboratory methods that can be replicated in resource-limited veterinary settings. However, the study has notable limitations. The exclusive use of classical parasitological techniques, while practical, limits sensitivity, particularly for protozoa and morphologically indistinguishable organisms such as *B. coli* and *B. sulcata*. Molecular confirmatory methods, which were not available, would refine species-level identification and strengthen zoonotic risk assessments. Additionally, reliance on breeder-reported deworming histories may have introduced recall bias.

Future research should prioritize the application of molecular diagnostics to characterize circulating parasite species and genotypes, particularly for *Cryptosporidium*, *Giardia*, and trematodes. Longitudinal studies are needed to clarify seasonal patterns, snail vector dynamics, and the impact of climate variability on parasite transmission. There is also a strong need for operational research to evaluate integrated parasite control programs, including anthelmintic rotation, targeted selective treatment, anticoccidial use, pasture rotation, and community-based farmer education. Expanding surveillance to neighboring districts will help build a broader epidemiological map of cattle parasitism in Mozambique under a One Health framework.

In conclusion, this study demonstrates that GI parasites remain a significant and under-recognized constraint to cattle health, productivity, and public health in the Limpopo district. By providing detailed baseline data and identifying key environmental and management-related risk factors, the findings offer a foundation for designing more effective, locally adapted parasite control strategies. Addressing these parasitic burdens is essential not only for improving livestock production but also for mitigating zoonotic risks and strengthening rural livelihoods in southern Mozambique.

## DATA AVAILABILITY

The supplementary data can be made available from the corresponding author upon request.

## AUTHORS’ CONTRIBUTIONS

ECM: Study design and conception, sample collection and processing, and manuscript writing. GAN: Statistical data analysis and manuscript review. ARM: Statistical data analysis and manuscript translation and review. ASM, IJM, and HC: Sample processing, manuscript review and language editing. BM: Statistical data analysis and manuscript review. CCM, LF, OMM, IS, PT, IMM, IJM, TD, and EMU: Sample collection and processing and manuscript writing. CA: Study design and conception, supervision, coordination of sample collection and processing, and manuscript writing and critical revision. All authors have read and approved the final version of the manuscript.
